# Supplement to the Account of the Good Effects of Opium in Mortifications

**Published:** 1784

**Authors:** 


V. *Supplement to the account of the good effects of Opium in mortifications. Communicated in a ſecond letter to Dr.* Simmons, F.R.S. *by* Robert Hamilton, M. D. *phyſician at Ipſwich, extra-licentiate*

*licentiate of the Royal College of Physicians, London; and member of the Medical Society of Edinburgh.*

AS a ſupplement to the account of the good effects of opium in mortifications, publiſhed in the London Medical Journal (vol. v. p. 75) I requeſt you to add, that Mr. Rolfe, the firſt patient alluded to, a farmer at Park-ſtreet near St. Albans, took no leſs than ſeven grains of ſolid opium at a doſe. In the ſpace of twenty-four hours he ſwallowed forty grains, with the addition of eighty drops of *tinct. theb.* in a pint of decoction of Peruvian bark.

In this caſe the mortification proceeded from a wound in the leg, upwards of ſix inches in length and four in breadth, received in attempting to open, and paſs through a gate on horſeback. If this plan had not been purſued he muſt have ſpeedily died, as the mortification had continued two days, and was ſpreading moſt rapidly, accompanied with a conſtant hiccup, and continual watching, attended with excruciating pain.

Mr. Law, who had the chief merit of this cure, gives the ſolid opium frequently in doſes of ten grains every two hours till reſt be obtained; tained; this is his criterion when to ſtop. What may appear to you ſingular (tho' he aſſures me it has often happened in his practice) is, that inſtead of producing conſtipation, which in ſmall doſes is the conſtant effect of opium; it proves in large doſes as certainly laxative. He farther declares, that he has adminiſtered opium in mortifications in this manner theſe thirty years.

